# Screening for oesophageal neoplasia in patients with head and neck cancer

**DOI:** 10.1038/sj.bjc.6600018

**Published:** 2002-01-21

**Authors:** H Scherübl, B von Lampe, S Faiss, P Däubler, P Bohlmann, T Plath, H-D Foss, H Scherer, A Strunz, B Hoffmeister, H Stein, M Zeitz, E-O Riecken

**Affiliations:** Medical Clinic I, University Hospital Benjamin Franklin, Free University of Berlin, Hindenburgdamm 30, 12200 Berlin, Germany; ENT Department, University Hospital Benjamin Franklin, Free University of Berlin, Hindenburgdamm 30, 12200 Berlin, Germany; Department of Maxillofacial Plastic Surgery, University Hospital Benjamin Franklin, Free University of Berlin, Hindenburgdamm 30, 12200 Berlin, Germany; Institute of Pathology, University Hospital Benjamin Franklin, Free University of Berlin, Hindenburgdamm 30, 12200 Berlin, Germany

**Keywords:** surveillance, secondary malignancy, squamous cell cancer, larynx, pharynx, oral cavity

## Abstract

Due to advanced disease at the time of diagnosis the prognosis of oesophageal cancer is generally poor. As mass screening for oesophageal cancer is neither feasible nor reasonable, high-risk groups should be identified and surveilled. The aim of this study was to define the risk of oesophageal cancer in patients with (previous) head and neck cancer. A total of 148 patients with (previous) head and neck cancer were prospectively screened for oesophageal cancer by video-oesophagoscopy and random oesophageal biopsies. Even in a macroscopically normal looking oesophagus, four biopsy specimens were taken every 3 cm throughout the entire length of the squamous oesophagus. Low- or high-grade squamous cell dysplasia was detected histologically in 10 of the 148 patients (6.8%). All but one dysplasias were diagnosed synchronously with the head and neck cancers. In addition, oesophageal squamous cell carcinoma was diagnosed in 11 of the 148 patients (7.4%). Most invasive cancers (63.6%) occurred metachronously. The risk of squamous cell neoplasia of the oesophagus is high in patients with (previous) head and neck cancer. Surveillance is recommended in this high-risk group.

*British Journal of Cancer* (2002) **86**, 239–243. DOI: 10.1038/sj/bjc/6600018 www.bjcancer.com

© 2002 The Cancer Research Campaign

## 

Heavy consumption of alcohol and tobacco is responsible for synchronous and metachronous primary malignancies in the upper aerodigestive tract. When a second oesophageal malignancy develops in a patient with previous head and neck cancer (HNC), the prognosis is determined by the oesophageal cancer, and unfortunately it is poor (Cooper *et al*, 1989; Leon *et al*, 1999). This is due to the advanced tumour stage at which oesophageal cancer becomes symptomatic. In patients with both HNC and oesophageal cancer, the former often develops first, and there are more metachronous than synchronous cases (Cooper *et al*, 1989; Makuuchi *et al*, 1996; Agrawal and Wenig, 1998; Leon *et al*, 1999). Screening for oesophageal squamous cell cancer has therefore been advocated for HNC patients (Cooper *et al*, 1989; Makuuchi *et al*, 1996; Horiuchi *et al*, 1998).

In analogy to the well-studied colorectal carcinogenesis, Mandard *et al* (2000) recently suggested a model of genetic steps in the development of squamous cell cancer of the oesophagus. The sequence of histopathological changes includes esophagitis, atrophy, mild to severe dysplasia, carcinoma *in situ* and, finally, invasive oesophageal cancer. The concept of a stepwise carcinogenesis implies the efficacy of surveillance in patients at high risk for squamous cell cancer of the oesophagus. Patients who survived HNC are known to develop a second neoplasm with a risk of 3–7% per year (Cooper *et al*, 1989; Sturgis and Miller, 1995). Prospective studies in Japan and recently in Brazil detected oesophageal squamous cell neoplasia in 5.1–11.8% of HNC patients (Shiozaki *et al*, 1990; Makuuchi *et al*, 1996; Tincani *et al*, 2000). General surveillance of HNC patients is not yet recommended in Western countries, mainly due to a lack of adequate data from this part of the world (Agrawal and Wenig, 1998; Deleyiannis and Weymuller, 1998).

In this study we used high-resolution video-oesophagoscopy to prospectively screen 148 HNC patients for oesophageal squamous neoplasia. To detect early histological changes even in a macroscopically normal looking oesophagus, we took multiple biopsies according to a systematic endoscopic biopsy protocol. The high rate of oesophageal neoplasia we observed confirms previous data from Japan and argues for a more general surveillance of HNC patients. As chromoendoscopy with Lugol staining only moderately improves the diagnostic accuracy of video-endoscopy (Meyer *et al*, 1997) in oesophageal squamous cell neoplasia, we applied Lugol chromoendoscopy only to assess the mucosal extension of early neoplastic lesions and to search for any additional neoplastic foci.

## MATERIALS AND METHODS

### Patients

A prospective study involving 148 patients with HNC was carried out at the University Hospital Benjamin Franklin in Berlin from May 2000 to August 2001. An oesophagogastroduodenoscopy with systematic oesophageal biopsies was suggested to all patients who presented with a previous history or first diagnosis of head and neck cancer (oral cavity, larynx, oro- or hypopharynx) to either the ENT Department, the Department of Maxillofacial Plastic Surgery or the Department of Gastroenterology. Patients with oesophageal varices or bleeding disorders were excluded. The study was approved by the Ethics Committee of the University Hospital Benjamin Franklin, Free University Berlin.

The 148 patients ranged in age from 34 to 89 years (average 61.0 years). Forty-two patients were women. The primary head and neck cancer was located in either the oral cavity (*n*=59), oropharynx (*n*=48), hypopharynx (*n*=23) or larynx (*n*=18 patients). In cases with multiple metachronous primary malignancies, the first head and neck carcinoma was regarded as the index tumour. When multiple head and neck tumours were diagnosed simultaneously, the tumour that caused the presenting complaint was chosen as the index lesion.

The oesophageal neoplasm was classified as synchronous if diagnosed within 6 months after the diagnosis of HNC and metachronous if diagnosed after a period of 6 months. Seventy-five of the 148 HNC patients were oesophagoscoped within 6 months after the diagnosis of HNC.

### Endoscopy

All endoscopies were performed using a high-resolution videoendoscopes (GIF-XQ 140, Olympus Optical Co. (Europa) GmbH, Hamburg, Germany). Biopsy specimens were obtained with the 7.5 mm open span biopsy forceps (K02 22 V-A, Endo-Flex, Voerde, Germany). Biopsy specimens were taken every 3 cm throughout the entire length of the squamous oesophagus, starting 3 cm above the gastrooesophageal junction. Four biopsies were taken from each level and placed in different bottles so that the locations could be separately documented. Even in a macroscopically normal looking oesophagus, 20 to 28 biopsies were sampled according to this systematic endoscopic biopsy protocol. In addition, multiple specimens were taken of any endoscopic abnormality.

### Histology

The final diagnosis was based on an independent review of the cases by two to three observers. If dysplasia was found, the microsections were evaluated by three different pathologists. All observers agreed on the diagnosis of cancer or dysplasia in the patients described in this study. In specimens where there was no complete agreement concerning the grade of dysplasia, two out of three was considered a consensus.

Oesophageal carcinomas if restricted to the submucosal layer were classified superficial and as intraepithelial if restricted to the epithelial layer (Endo *et al*, 1986; Sugimachi *et al*, 1988; Mori *et al*, 1993; Schlemper *et al*, 2000c). Low- and high-grade dysplasia (synonym: intraepithelial neoplasias; Gabbert *et al*, 2000) was defined as unequivocal neoplastic transformation according to criteria published previously (Lewin and Appelman, 1995).

## RESULTS

### Oesophageal squamous cell cancer (ESCC)

Among the 148 patients examined, 14 invasive oesophageal squamous cell cancers were detected in 11 patients (7.4%; Table 1

**Table 1:**
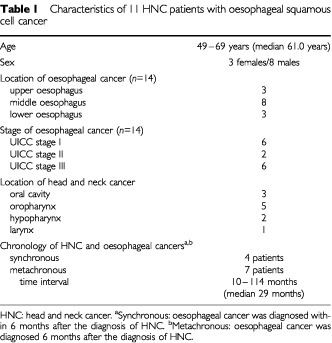
Characteristics of 11 HNC patients with oesophageal squamous cell cancer

). Three of the 11 patients even had two oesophageal cancers each; in addition to two oesophageal cancers two of the three patients suffered from oesophageal dysplasia at a separate third location. In four of the 11 patients, HNC and oesophageal cancer were diagnosed synchronously. Interestingly, the patients related their clinical symptoms only to the head and neck region. The other seven patients had metachronous oesophageal cancers that developed 10, 13, 27, 29, 41, 74 and 114 months after the (first) head and neck cancer. These patients did not present with dysphagia. The oesophageal cancers were preceded by 15 primary head and neck cancers (mean of 1.4 HNC per ESCC-patient). In contrast, the HNC patients in whom no oesophageal dysplasia or cancer was detected had suffered from a mean of 1.1 HNC per patient.

Apart from the 148 study patients, seven further HNC patients presenting with dysphagia during the study period were diagnosed as suffering from squamous cell cancer of the oesophagus. Since these seven patients complained of dysphagia, they were not included in the group of 148 study patients. All seven patients suffered from stenotic stage III squamous cell cancer of the oesophagus. The intervals between the diagnosis of (the first) HNC and oesophageal cancer were 2, 3, 5, 7, 8, 9 and 13 years. The seven patients had developed 10 HNC prior to the now presented oesophageal cancers.

### Oesophageal squamous cell dysplasia (ESCD)

Even in a macroscopically normal looking oesophagus we sampled multiple biopsies according to the outlined systematic endoscopic biopsy protocol. Oesophageal squamous cell dysplasia (eight low- and four high-grade dysplasias) was detected in 10 of the 148 (6.8%) patients biopsied according to that protocol (Table 2

**Table 2:**
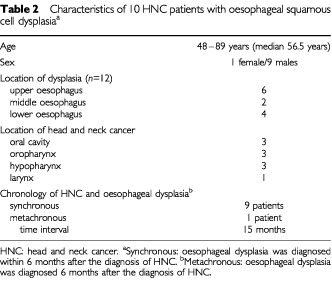
Characteristics of 10 HNC patients with oesophageal squamous cell dysplasia^a^

). In nine of the 10 patients, HNC and squamous cell dysplasia (= intra-epithelial neoplasia) of the oesophagus were diagnosed synchronously. Altogether 13 head and neck cancers had occurred in the 10 patients with oesophageal dysplasia (mean 1.3 HNC per patient). All patients found to suffer from dysplasias or early oesophageal cancer were further examined by chromoendoscopy with Lugol dye solution (Shiozaki *et al*, 1990; Endo *et al*, 1986; Sugimachi *et al*, 1988) and by endoscopic ultrasound (Ziegler *et al*, 1991). Lugol chromoendoscopy identified all but one dysplastic lesions and proved very helpful in assessing the mucosal extension of early neoplastic lesions (Figure 1

**Figure 1:**
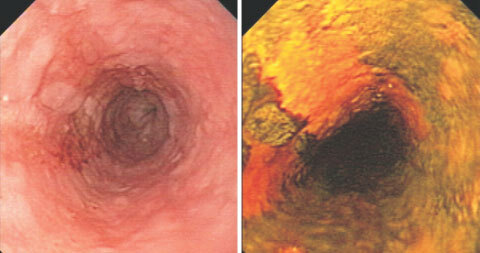
Oesophageal squamous cell cancer in a HNC patient. Left panel: Videoendoscopic image of a T_1_N_o_ squamous cell cancer at 25 cm from the incisors. Twenty-nine months ago the patient had been treated for a squamous cell cancer of the oral cavity. Right panel: The same tumour after staining with Lugol dye solution to delineate the tumour margins.

); additional neoplastic foci were not detected by Lugol staining. There were no major complications related to the endoscopic examinations or the biopsies taken.

## DISCUSSION

Oesophageal cancer is a disease with a dramatically different geographic incidence. In high-risk areas like Linxian, China, mass surveillance programmes for the malignancy have been implemented to reduce the high mortality rate of 161 per 100 000 (Wu *et al*, 1982). In most Western countries, the incidence of oesophageal squamous cell cancer is less than 6 per 100 000 (Fleischer and Haddad, 1999; Gabbert *et al*, 2000). The rarity of this neoplasm does not justify implementing a costly endoscopic surveillance programme for the general population in low-risk areas. However, even in low-risk areas, surveillance may be warranted for high-risk groups. High-risk groups for oesophageal squamous cell cancer include male heavy drinkers and smokers, patients with corrosive esophagitis, achalasia or with (previous) head and neck cancer (Makuuchi *et al*, 1996).

The incidence of a second neoplasm in patients who survive HNC is 3–7% per year (Cooper *et al*, 1989; Sturgis and Miller, 1995; Ina *et al*, 1994). Nevertheless, surveillance of HNC patients has thus far not been generally recommended. This is mainly due to a lack of adequate data, the difficulty of early diagnosis and the lack of safe and effective therapeutic options (Agrawal and Wenig, 1998; Deleyiannis and Weymuller, 1998). Here we provide evidence that 11 patients out of a group of 148 patients with (previous) HNC suffered from oesophageal squamous cell cancer. This high frequency may have been biased by the referral practice to our hospital which provides care for HNC patients in a population of about 750 000 people in the southwestern part of Berlin. In referral centers in Germany, as many as 25–35% of the newly diagnosed oesophageal squamous cell cancers of the oesophagus are now reported in HNC patients. Many patients with previous HNC may be used to some degree of odyno- or dysphagia so that they are hardly alert to any symptoms of early oesophageal cancer. This may explain why oesophageal cancer is generally diagnosed late even in patients who have already experienced the sequelae of HNC (Cooper *et al*, 1989; Leon *et al*, 1999). Thus, in our study, six of the 11 patients with oesophageal cancer already had a stage III tumour disease.

In order to detect early lesions even in a macroscopically normal looking and staining oesophagus (Fagundes *et al*, 1999), we took multiple biopsies according to a systematic endoscopic biopsy protocol and found that 6.8% (10 out of 148) of the patients biopsied had oesophageal squamous cell dysplasias (synonym: intraepithelial neoplasias; Gabbert *et al*, 2000; Schlemper *et al*, 2000a, 2000b, 2000c). This high percentage compares well or even surmounts the rates observed in previous studies (Makuuchi *et al*, 1996; Meyer *et al*, 1997; Fagundes *et al*, 1999; Tincani *et al*, 2000; Petit *et al*, 2001) and provides strong evidence for the efficacy of the screening protocol used in this study. Various alternative screening strategies have been described. These include brush-capsule cytology (Leoni-Parvex *et al*, 2000), biannual oesophageal endoscopy without Lugol staining (Petit *et al*, 2001), Lugol chromoendoscopy with (Fagundes *et al*, 1999) or without random biopsies (Shiozaki *et al*, 1990; Makuuchi *et al*, 1996), (auto)fluorescence endoscopy (Haringsma *et al*, 2001) or tri-modal spectroscopy (Georgakoudi *et al*, 2001). These different strategies have, however, not yet been compared in controlled clinical trials.

Due to its low price and general availability Lugol staining has probably been used most commonly. Drawbacks of Lugol staining are the low yield of neoplasia in small unstained areas (<1% ESCC risk in unstained areas of <5 mm) and the missing of normal staining intra-epithelial neoplasias. By taking two random biopsies in the middle of the oesophagus, Fagundes *et al* (1999) observed intra-epithelial neoplasias in 7 out of 165 (4.2%) alcoholic smokers despite a normal Lugol stain of the squamous oesophagus. Therefore, randomized controlled studies are indicated to compare the efficacy and cost effectiveness of the various strategies.

Since mucosal surfaces in the upper aerodigestive tract, lungs, and the oesophagus are exposed to the same carcinogens, multiple anatomical sites in the oesophagus may be at risk for the simultaneous or sequential development of dysplastic and malignant lesions. This multifocal large-field carcinogenesis is reflected by the finding that 11 patients with oesophageal cancer had suffered from a mean of 1.4 primary HNC, 10 patients with oesophageal dysplasia from a mean of 1.3 HNC and the remaining patients from a mean of 1.1 primary HNC. This suggests that patients with multiple HNC have an even higher risk of developing or already harbouring oesophageal dysplasia or cancer. Oesophageal cancer was multifocal in 27.3% of our patients. In addition, we observed a similar rate of oesophageal dysplasia and oesophageal squamous cell cancer (6.8 *vs* 7.4%). All but one oesophageal squamous cell dysplasias were detected synchronously, but cancer was mostly (63.6%) diagnosed metachronously.

Thus, our findings imply that squamous cell dysplasia precedes oesophageal squamous cell cancer. This supports the concept of a stepwise development of oesophageal squamous cell cancer recently put forward by Mandard *et al* (2000). Although most second oesophageal squamous cell cancers appear to develop as independent neoplasms, it has recently been reported that a clonal population of neoplastic cells from the oro- or hypopharynx may be capable of travelling substantial distances to give rise to second tumours in the oesophagus (Califano *et al*, 1999).

The introduction of endoscopic oesophageal mucosal resection (EEMR) has revolutionized the treatment of intra-epithelial oesophageal neoplasia and early cancer (Makuuchi, 1996; Ell *et al*, 2000). In experienced hands, this therapeutic endoscopic technique has a very low morbidity and mortality and is an attractive alternative to oesophageal resection in certain situations (Makuuchi *et al*, 1996; Horiuchi *et al*, 1998; Ell *et al*, 2000). The more general availability of EEMR offers HNC patients not only an earlier diagnosis of oesophageal cancer but also an effective minimal invasive treatment option if oesophageal squamous cell neoplasia is diagnosed early. Photodynamic therapy (with haematoporphyrins) has also been reported to eradicate early squamous cell cancer in HNC patients (Savary *et al*, 1998). Thus, surveillance of oesophageal squamous cell cancer is recommended in HNC patients (Makuuchi *et al*, 1996). In addition, the survival benefit achieved by screening for early oesophageal cancer (Horiuchi *et al*, 1998) underlines the need for a more general implementation of surveillance in HNC patients.
